# Drought-modulated allometric patterns of trees in semi-arid forests

**DOI:** 10.1038/s42003-020-01144-4

**Published:** 2020-07-30

**Authors:** Jingyu Dai, Hongyan Liu, Yongcai Wang, Qinghua Guo, Tianyu Hu, Timothy Quine, Sophie Green, Henrik Hartmann, Chongyang Xu, Xu Liu, Zihan Jiang

**Affiliations:** 1 College of Urban and Environmental Science and MOE Laboratory for Earth Surface Processes, Peking University, 100871 Beijing, China; 2 Satellite Environmental Application Center, Ministry of Ecology and Environment, 100094 Beijing, China; 3 State Key Laboratory of Vegetation and Environmental Change, Institute of Botany, Chinese Academy of Sciences, 100093 Beijing, China; 4 Department of Geography, University of Exeter, Exeter, EX4 4RJ UK; 5 Max Planck Institute for Biogeochemistry, 07745 Jena, Germany; 6 Urban-Rural Planning Administration Center, MOHURD, 100871 Beijing, China; 7 Department of Natural Resources, Institute of Temperate Forest Sciences and Centre for Forest Research (CEF), Université du Québec en Outaouais, 58 Rue Principale, Ripon, QC J0V 1V0 Canada

**Keywords:** Ecology, Plant ecology

## Abstract

Tree allometry in semi-arid forests is characterized by short height but large canopy. This pattern may be important for maintaining water-use efficiency and carbon sequestration simultaneously, but still lacks quantification. Here we use terrestrial laser scanning to quantify allometry variations of *Quercus mongolica* in semi-arid forests. With tree height (Height) declining, canopy area (CA) decreases with substantial variations. The theoretical CA-Height relationship in dynamic global vegetation models (DGVMs) matches only the 5^th^ percentile of our results because of CA underestimation and Height overestimation by breast height diameter (DBH). Water supply determines Height variation (*P* = 0.000) but not CA (*P* = 0.2 in partial correlation). The decoupled functions of stem, hydraulic conductance and leaf spatial arrangement, may explain the inconsistency, which may further ensure hydraulic safety and carbon assimilation, avoiding forest dieback. Works on tree allometry pattern and determinant will effectively supply tree drought tolerance studying and support DGVM improvements.

## Abstract (web summary)

Jingyu Dai et al. study the role of tree allometry in maintaining a high carbon sequestration. They measure allometry in semi-arid forests using terrestrial laser scanning. They report that tree height as well as canopy area decline to varying degrees among individuals. They provide insights on how trees balance water consumption and carbon assimilation which enhances the survival rate of the forest trees.

## Introduction

The short and open forests in semi-arid regions account for 57% and 39% of the global terrestrial CO_2_ sink trend and interannual variation, respectively^1,2^. Despite the fact that these forests receive only 1/3 of the global mean annual precipitation^3^, the gross primary productivity of semi-arid forests can reach 8.2 tons C ha^−1^, which is approximately 72% and 53% of the carbon sequestration in European forests and the entire global Fluxnet network^4^, showing a significantly higher water-use efficiency than that in other ecosystems^5^. It is worth understanding how the “short trees” bear a high water-use efficiency and carbon sequestration simultaneously in semi-arid regions, ~17.7% of the total land surface area^4,6^.

Forestry surveys have revealed that the allometric relationships among tree height (Height), canopy area (CA) and breast height diameter (DBH) are mainly driven by light limitations^7^. However, in open forests such as semi-arid forests, light is generally not a limiting factor for tree growth. The dry climate found in semi-arid regions may limit the height growth of trees^8^, thereby creating an extreme allometric pattern with short trees but wide canopy. Allometric growth modules of many dynamic global vegetation models (DGVMs), however, do not take this special Height-CA-DBH relationship of dry forests into consideration. For example, in one of the earliest DGVMs, the Lund-Potsdam-Jena (LPJ) model, the allometric relationships between Height and DBH and between CA and DBH were established only by data collected from the Rocky Mountains region in North America with a relatively humid climate^9,10^. Furthermore, the curves relate the allometric pattern only to tree density under self-thinning conditions^9^. The allometric growth equations Height = 40 × DBH^0.5^ and CA = 100 × DBH^1.6^ used in LPJ^9,10^ can be combined into a theoretical model of the CA-Height relationship (CA = 0.000747 × Height^3.2^), implying a steeper decrease in tree canopy size with tree height reduction compared with the actual trees in semi-arid forests. Therefore, models based on these relationships are incorrect for simulating vegetation carbon sequestration potential of semi-arid regions. What’s more, almost all the DGVMs with allometric growth module chose to apply the one in LPJ directly or adjusted the parameters slightly. For example, the DGVMs that used the same growth module with LPJ including the Community Climate System Model^11^, the Community Land Model’s Dynamic Global Vegetation Model^12^, O-CN land surface model^13^ and the Organizing Carbon and Hydrology In Dynamic Ecosystems^14^, while DGVMs that made slightly adjustments on parameters including the Community Atmosphere–Biosphere Land Exchange model^15^, Hybrid3^16^, HyLand^17^, the IAP Dynamic Global Vegetation Model^18^, LPJ-GUESS^19^ and the spatially explicit individual-based Dynamic Global Vegetation Model^20^. Therefore, all the involving DGVMs may have systematic bias for semi-arid forests carbon sequestration potential due to the simplistic allometric growth module.

Among the three allometric traits studied herein, DBH represents the tree carbon accumulation and growth processes over years^21,22^, while Height and CA are more likely to have plastic responses and acclimate to the environment. Height and CA are tightly linked to tree hydraulic conductance^23–25^ and leaf spatial arrangement for light harvesting^26^, respectively. Tree hydraulic conductance and leaf spatial arrangement are two of the most important functions of stem. Hydraulic conductance is related to plant water utilization^24^, while leaf spatial arrangement determines the carbon sequestration potential^27^. A more effective and flexible combination of the two stem functions may provide the possibility for trees in semi-arid forests to keep high water-use efficiency and carbon sequestration simultaneously. The architecture plasticity may therefore reduce the risk of semi-arid forests to dieback due to hydraulic failure or carbon starvation^28^. Meanwhile, a more mechanistic implementation of CA-Height responses to environmental limiting factors in forests, especially water deficit under dry climate, can provide a theoretical basis for improving DGVM simulations in semi-arid area.

However, few studies have investigated large-scale tree morphology and allometric patterns due to the lack of suitable methods^12,29^. It is only recently that terrestrial laser scanning (TLS) has provided a field-applicable and affordable solution for such studies^23^. TLS makes it possible to characterize the three-dimensional features of trees using active remote sensing technology (i.e., light detection and ranging, LiDAR)^30^. Ground-based LiDAR scanner systems provide accurate measurements of an object’s distance to the scanner position^31^ and allows quantifying almost all tree morphological traits, including tree height^21^, crown volume^32^, and branch angle^33^, and thus permits computing community biomass^34^. TLS is now widely used in regional carbon pool estimates and species coexistence studies^35^. One can carry out tree morphology measurements repeatedly with TLS to get more reliable and reproducible data^36^.

We measured several tree morphological traits, including the allometric traits Height, CA, and DBH, over a large geographical area. We hypothesize that tree allometry shifts towards smaller stature and wider canopies with increasing water limitation in semi-arid regions, and the tree allometry shifts can be explained by the flexible combination of the two stem functions, hydraulic conductance and leaf spatial arrangement. We test this hypothesis on *Quercus mongolica*, a dominant species in East Asian semi-humid and semi-arid regions, by quantifying tree morphological variation along an environmental gradient, revealing the relationships among traits and between traits and the environment. We focus on the Height-CA-DBH relationship in semi-arid regions firstly, to test whether it can be represented by the theoretical relationships widely adopted by DGVMs. Then, we test the relationship among tree morphological traits related to hydraulic conductance and leaf spatial arrangement, and the relationship between plant morphological traits and environment factors, to see if the stem functions relationship can be the reason of the observed allometry pattern in semi-arid forests.

## Results

### The observed and theoretical tree allometry relationships

**Fig. 1:**
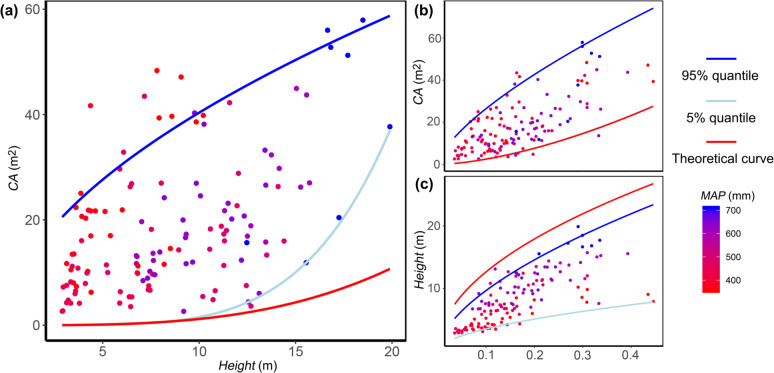
Relationship between individual-level tree height (Height), canopy area (CA) and breast height diameter (DBH) and their comparisons with the theoretical curves. The colors of the dots show the mean annual precipitation (MAP) where the trees are located. The nonlinear 95% and 5% quantile regression curves (*n* = 133) are shown in dark and light blue, respectively, while the theoretical curves are shown in red. The formulas of the nonlinear regressions are as follows: (**a**) 95% quantile: CA = 11.48 × Height^0.546^, 5% quantile: CA = 3.73 × 10^−5^ × Height^4.62^, and theoretical: CA = 7.5 × 10^−4^ × Height^3.2^; (**b**) 95% quantile: CA = 129.2 × DBH^0.69^, 5% quantile: CA = 99.35 × DBH^1.58^, and theoretical: CA = 100 × DBH^1.6^; (**c**) 95% quantile: Height = 37.62 × DBH^0.59^, 5% quantile: Height = 12 × 10^−8^ × DBH^0.53^, and theoretical: Height = 40 × DBH^0.5^.

For the investigated *Q. mongolica*, CA and Height showed a positive correlation (CA = 2.068 × Height, *P* = 0.000). However, the upper and lower bounds (95^th^ and 5^th^ percentiles, respectively) of CA showed distinct trends, in which the upper bound followed the formula CA = 11.48 × Height^0.546^ and the lower bound followed the formula CA = 3.73 × 10^−5^ × Height^4.62^ (Fig. 1a).

The allometric growth equations Height = 40 × DBH^0.5^ and CA = 100 × DBH^1.6^ used in DGVMs can be combined into an equation representing the CA-Height relationship (CA = 7.5 × 10^−4^ × Height^3.2^), which results in a value lower than the minimum CA in our results (i.e., the 5th percentile). The difference between the theoretical and minimum CA-Height curves becomes larger as Height increases (Fig. 1a).

We found similar pattern between the theoretical and observed CA*-*DBH relationships: the theoretical curve, CA = 100 × DBH^1.6^, was consistent with the curve of our 5% quantile nonlinear regression equation, CA = 99.35 × DBH^1.58^ (Fig. 1b). Theoretical curve of the Height-DBH relationship, Height = 40 × DBH^0.5^, is generally higher than the curve of the 95% quantile nonlinear regression, the equation of which was Height = 37.62 × DBH^0.59^ (Fig. 1c).

### Statistical relationships among tree morphological traits

Pearson correlation and partial correlation tests revealed relationship among the stem and leaf morphological traits (Supplementary Fig. 1). Height, CA, and DBH were all positively correlated with each other in correlation test (*P* = 0.000), while Height and CA were negatively correlated in the partial correlation tests (*P* = 0.030). Height showed a significant correlation with all morphological traits, either positive or negative (*P* < 0.050), except the ratios of the second-order and first-order branch lengths (Sl/Fl) (*P* = 0.274). For the partial correlation tests, only specific leaf area (SLA) was negatively correlated with Height (*P* = 0.073) in addition to DBH and CA, as previously mentioned. The relationship between CA and the other morphological traits had smaller correlation coefficients and are more varied than the relationship between Height and the other morphological traits. CA showed a significant negative correlation with leaf area index (LAI) (*P* = 0.005) and leaf tissue density (LTD) (*P* = 0.038), and a positive correlation with SLA (*P* = 0.013), leaf area (LA) (*P* = 0.021) and leaf main vein length (LV) (*P* = 0.020) in the correlation tests. Meanwhile, CA was negatively correlated with the ratio of height under the crown and tree height (CLR) (*P* = 0.045) but positively correlated with LTD (*P* = 0.013) and SLA (*P* = 0.015) simultaneously in the partial correlation tests. The results pointed out the robust positive correlation between Height and SLA, while the relationship between CA and leaf traits cannot be summarized.

**Fig. 2:**
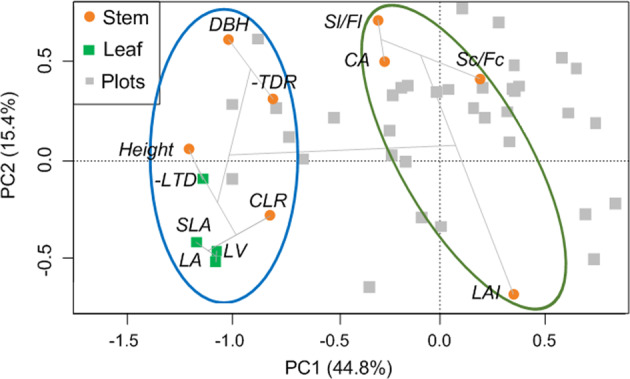
Images showing the patterns of the stem and leaf morphological traits. The stem and leaf morphological traits are shown as orange dots and green squares, respectively. The gray squares indicate the PCA scores for the 36 plots. The cluster analysis results are shown as gray lines (*n* = 36). The abbreviations are as follows: plant height (Height), ratio of the height under the crown and tree height (CLR), breast height diameter (DBH), trunk dominance ratio (TDR), canopy area (CA), leaf area index (LAI), ratio of the second-order and first-order branch lengths (Sl/Fl) and counts (Sc/Fc), leaf area (LA), specific leaf area (SLA), leaf tissue density (LTD), and leaf main vein length (LV). For a detailed explanation of the plant traits, refer to Supplementary Table 3.

The principal components extracted from morphological traits of *Q. mongolica* (Fig. 2) explained 60.2% of the total variance, 44.8% by the first and 15.4% by the second axis. Leaves and some of the stem morphological traits were associated with the first axis, including Height, CLR, LTD and the trunk dominance ratio (*TDR*), which we created to describe whether a tree had a single, dominant trunk or was shaped like a shrub with multiple basal stems (see Methods and Supplementary Fig. 2 for details). Other stem morphological traits, including Sl/Fl, Sc/Fc, LAI, and CA, were mainly associated with the second axis. In the cluster analysis, Sl/Fl, Sc/Fc, LAI, and CA were clearly distinguished from the other traits (Fig. 2). This pattern indicated that variations in stem morphological traits fell into two orthogonal categories: Height, CLR, LTD, and TDR varying coupled with leaf morphological traits, while CA, Sl/Fl, Sc/Fc, and LAI were decoupled with leaf morphological traits. As the leaf morphological traits have been proved to be tightly related with leaf water use strategies (Supplementary Fig. 3). The results of PCA and cluster analysis revealed that the variations of Height, CLR, LTD, and TDR were related to the stem hydraulic transportation function, while CA, Sl/Fl, Sc/Fc, and LAI were not.

### Environmental impacts on tree morphological traits

**Fig. 3:**
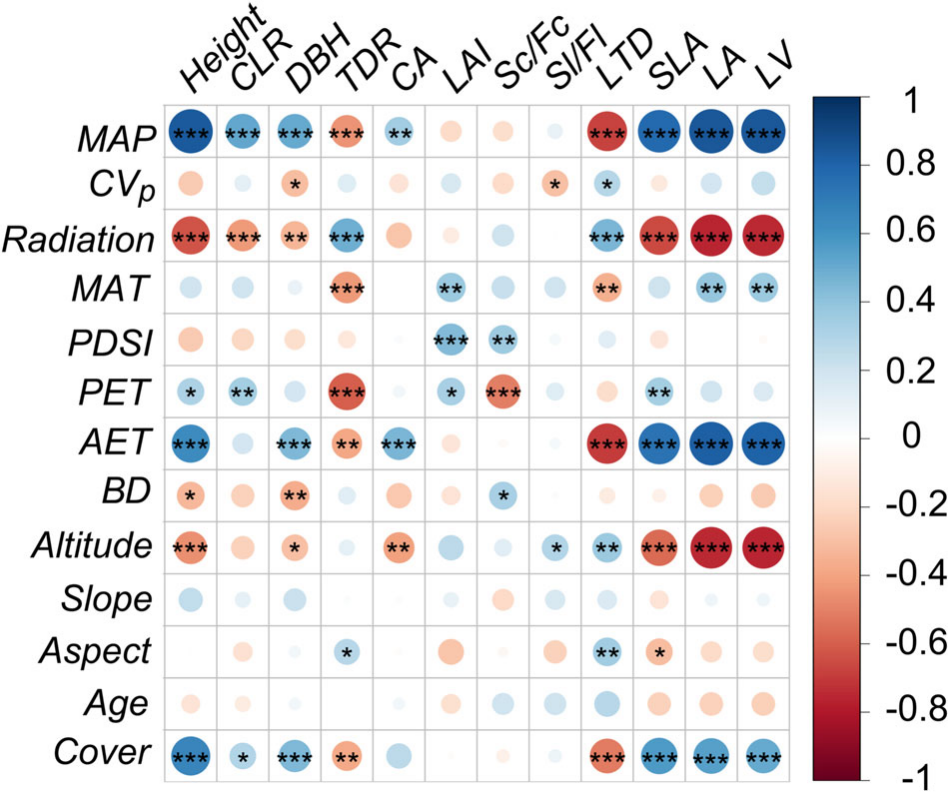
Results from the two-tailed Pearson correlation analysis between the environmental factors and tree morphological traits (*n* = 36). The color and size of the circles show the correlation coefficient. The asterisk indicates significance: *P < 0.1; **P < 0.05; ***P < 0.01. The abbreviations used for the plant traits and environmental factors are as follows: plant height (Height), ratio of the height under the crown and tree height (CLR), breast height diameter (DBH), trunk dominance ratio (TDR), canopy area (CA), leaf area index (LAI), ratio of the second-order and first-order branch lengths (Sl/Fl) and counts (Sc/Fc), leaf area (LA), specific leaf area (SLA), leaf tissue density (LTD), and leaf main vein length (LV); mean annual precipitation (MAP), interannual variability in precipitation (CV_P_), regional solar irradiation (Radiation), mean annual temperature (MAT), Palmer drought severity index (PDSI), annual potential evapotranspiration (PET), annual actual evapotranspiration (AET), soil bulk density (BD), altitude (Altitude), slope (Slope), aspect (Aspect), average tree age (Age), and percentage tree cover (Cover) of the plots. For a detailed explanation of the plant traits, refer to Supplementary Table 3.

Environmental factors could be divided into three categories according to their impacts on plant traits: those having the same influence with mean annual precipitation (MAP), with regional solar irradiation (Radiation), and those that had little impact on the tree morphological traits. The first category was composed of MAP, annual actual evapotranspiration (AET), annual potential evapotranspiration (PET), mean annual temperature (MAT), and percentage tree cover (Cover), while the second category consisted of Radiation, the altitude of the plots (Altitude), the Palmer drought severity index (PDSI), soil bulk density (BD), and plot aspect (Aspect). These two categories had opposite impacts on tree allometry and morphological traits, while within each category, the factors tended to have similar impacts on the tree morphological traits, but with varying levels of significance. Interannual variability in precipitation (CV_P_), plot slope (Slope) and average tree age (Age) had little impact on the tree morphological traits (Fig. 3).

With increasing Radiation, Height (*P* = 0.000), CLR (*P* = 0.009), and DBH (*P* = 0.043) significantly decreased, while TDR significantly increased (*P* = 0.002). Regarding leaf morphological traits, LA, SLA, and LV decreased, while LTD increased with increasing Radiation (*P* = 0.005). The other stem morphological traits had neutral responses to varied Radiation, including CA, LAI, Sc/Fc, and Sl/Fl (*P* > 0.1, Supplementary Fig. 4).

When removing the effects of the environmental factors on Height and CA through affecting tree biomass accumulation (indicated with DBH, Supplementary Table 1), the significance declined strongly between CA and MAP, AET, Altitude compared with the correlation tests, in which MAP-CA and AET-CA became statistically insignificant (for MAP-CA: *P* = 0.154, for AET-CA: 0.371, and for Altitude-CA: *P* = 0.057). Similar significance declines occurred for AET-CA and Altitude-CA relationships when Height was controlled for (*P* = 0.486 and 0.292, respectively). In contrast, correlations between Height and the environmental factors were all robust, regardless of whether DBH or *CA* were controlled (*P* < 0.05 for most tests, *P* = 0.098 for the Altitude-Height relationship when CA was controlled for). The results revealed that the linkage between Height and regional water supply factors were robust, while the linkage between CA and environment water supply were achieved through Height and DBH.

## Discussion

Our results showed that with Height declining, CA gradually decreased with very large variations across individuals. The theoretical CA-Height curve adopted by most DGVMs matched only the 5^th^ percentile of our results, the minimum CA for a given Height, with gradually increasing underestimation as Height increased. This overall underestimation could be attributed to the underestimation of CA and overestimation of Height by DBH in DGVMs. The small-CA individuals occurred generally in the denser parts of the communities, which could be attributed to light and space limitations in competition among individuals. The theoretical allometry relationship reflects exactly the light and space limitations of trees in humid forests under self-thinning conditions^9,37^, which may occur in less than 5% of our data, while the impact of other environmental limitations on tree allometry, in particular aridity, has been overlooked.

A possible explanation for the special Height-CA allometric patterns in semi-arid forests may be the decoupled stem functions, hydraulic conductance and leaf spatial arrangements. Smaller tree stature implies shorter water transport pathways and reduce the differences in water potential between leaves and roots in water-limited areas^24,38,39^, thus adjusting hydraulic traits and safety margin^25,40^. Reduced CA may also make difference on shortening hydraulic paths, but the loss of CA will lead to a disadvantage for trees in the balance between carbon sequestration and hydraulic safety maintain in water-limited systems. On the one hand, the effect of reducing CA on adjusting hydraulic safety is not as important as reducing tree height, because the vertical pathlength in trunk implies a much higher water stress due to gravity than the horizontal pathlength in crowns. On the other hand, reduced CA would cause a simultaneous decrease of the tree light harvesting area and light gain potential following a quadratic function^26,27,41^. The decoupled Height-CA relationship secure tree hydraulic integrity while preventing trees from encountering a light-harvesting limitation, thus maintaining the carbon assimilation potential of forests. The flexible allometric pattern of the trees may thus partly decrease the risk of forest mortality caused by hydraulic failure or carbon starvation^28^, which would further benefit semi-arid forest sustainability and carbon sink potential with climate changing.

Our results show that the theoretical underpinnings and environmental limitations in tree allometry modeling must be revised, as light limitation is generally replaced by increasing water stress in drought-modulated ecosystems, such as semi-arid forests^8^. In fact, trees with low Height but large CA are important ecosystem components in many regions: mallee scrubland in Australia is dominated by the shrub-like eucalyptus tree species (*Eucalyptus* spp.)^42^, in South Africa *Acacia karroo* takes the shape of a wide-canopy shrub in arid shrublands, which can grow into tall trees in humid forests^43^, and some other broadleaf trees like birch (*Betula platyphylla*) in semi-arid areas show the same shrubby shape in North Asia (see Fig. 2 of ref. ^44^). Environmental factors other than light and rainfall can also shape trees. For example, tree architecture of *A. karroo* in savannas and arid shrublands is shaped to maximize avoidance of damage by fires and grazing, respectively^43^; wind can also shape tree architecture to some extent^45^. To better describe tree allometry in these non-light-driven ecosystems, it is necessary to add a threshold for the DBH-Height-CA relationships based on the dominant regional environmental factors in addition to the light-limited growth-driven allometry formulas typically used in DGVMs.

In summary, the long-term water limitations in semi-arid regions has caused trees to form a special allometric pattern with short tree height but large canopy areas. The decoupled relationship between the functions of stem, hydraulic conductance and leaf spatial arrangements, is a possible explanation for the allometric pattern in semi-arid forests, and might be an important strategy for trees to survival at the driest edge of forest distribution, which is becoming even drier with climate change. There is a large discrepancy between observed and theoretical allometric growth relationships implemented in DGVMs, where short trees have very small canopies based on the DBH-driven Height-CA relationship. This discrepancy could lead to a systematic bias on allometric modeling of trees and, therefore, on modeling tree carbon assimilation potential in DGVMs. With the unrealistic tree allometric relationships, DGVMs are widely used in spatial comparisons and regional vegetation dynamic forecasting^46,47^, and an underestimation of carbon sequestration in semi-arid regions has been documented^48^. However, our regional assessment of environmental impacts on allometric relationships in semi-arid forests has a limited representability of global patterns, thus we still cannot provide the proper revision for the DGVMs. A systematic mapping of the Height-CA-DBH relationship and determinants on the global scale, and a mechanism based revision for the allometric module in DGVMs are required for the future works.

## Methods

### Study area

**Fig. 4:**
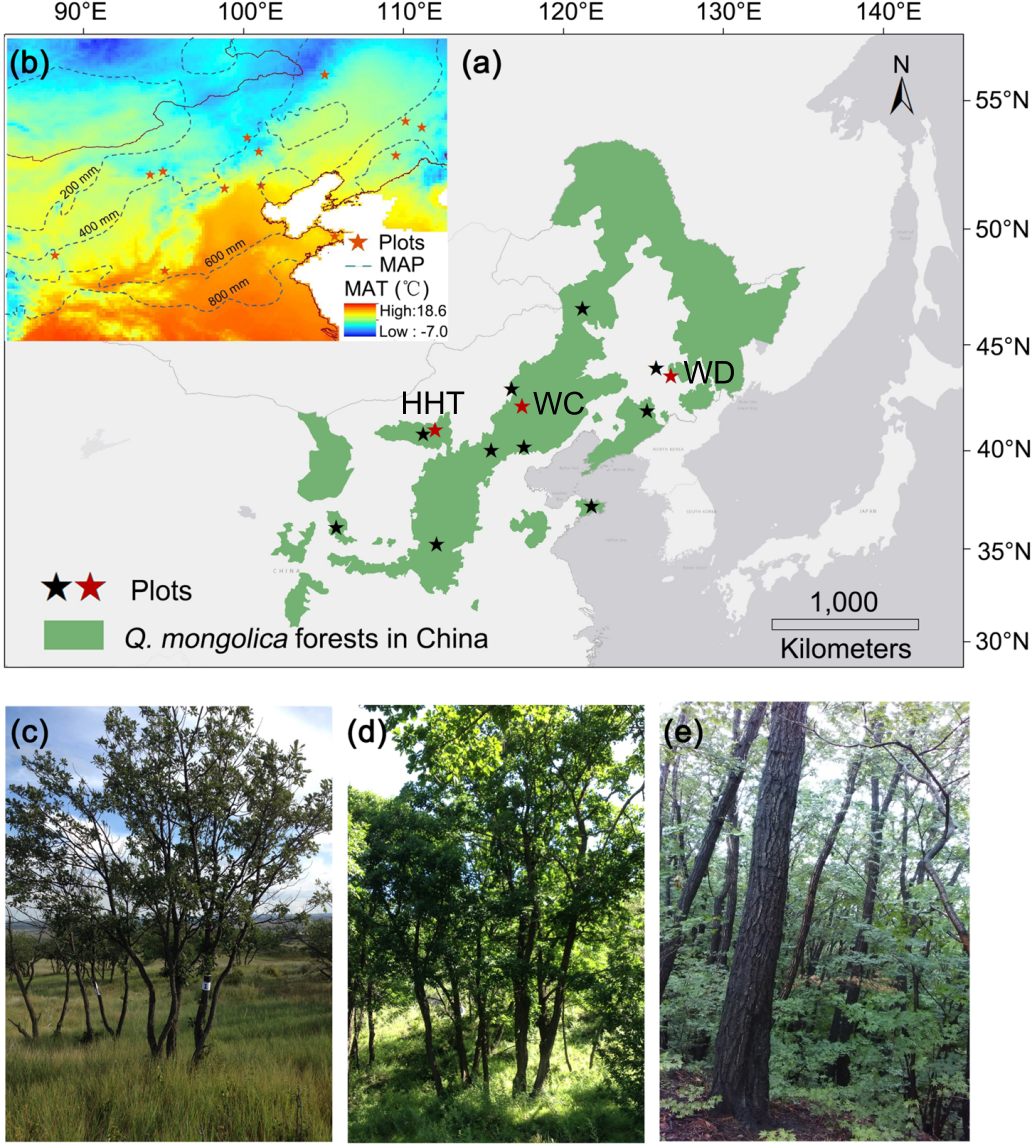
Study area and location of sampling plots. **a** The location of the study area, showing the sampling plots and the distribution of *Quercus mongolica*. The green shading indicates the natural distribution of *Q. mongolica* forest in China, according to ref. ^61^. The asterisks mark the sampling plots. **b** The subpanel shows the mean annual temperature (MAT) of the study area with different colors, while the mean annual precipitation (MAP) is shown by the isohyet. The photos below show tree morphological changes along the geographical gradient, taken from the (**c**) Hohhot (HHT), (**d**) Weichang (WC), and (**e**) Wandian (WD) plots (marked in subfigure **a** with red asterisks) in the summer of 2015.

The study area is situated in the semi-humid and semi-arid areas in northern China (Fig. 4), in the southwestern and driest part of the global distribution of *Q. mongolica*^49^. The MAP ranges from approximately 350–800 mm, and MAT is 1.5–14.5 °C according to the WorldCLIM dataset^50^. The study area is located in the ecotone of the temperate monsoon and continental climate. The characteristics of surface wind among the sites are homogeneous, which minimizes the possible effects of wind intensity variation on tree architecture^45^.

Twelve sites along a MAP gradient with different MAT levels were chosen for sampling in northern China in 2015 and 2016 (Supplementary Table 2, Fig. 4). Three 25 × 25 m plots were established at each study site. Pure and mature *Q. mongolica* forests with tree densities as low as possible were chosen to reduce the influence of inter-specific and intra-specific competition. No obvious evidence of fire, insect attack, cutting or grazing was detected in the plots selected for study.

### Sampling design

In each plot, geographic information, including latitude, longitude, altitude, slope and aspect, was recorded. The Riegl VZ-400 terrestrial LiDAR system (Riegl, Austria) was used to capture stem morphological features. Ten to thirteen scan stations were established to ensure that all of the trees in each plot were measured. For plots with tree heights over 10 m, the scanning was performed twice to ensure that the canopy was scanned intact, horizontally and 30° upward. The percentage tree cover of the plots was calculated with point clouds and the methods recorded in the ref. ^51^.

Leaves were sampled from both sunny and shady branches at middle and lower height of tree canopy. The leaves were scanned in the field using a portable scanner (Founder *MobileOffice* Z6, Founder, China). Meanwhile, leaf thickness was measured using a slide calliper.

Two cores were taken from eight trees per plot, with the trees being selected randomly from those with a DBH > 5 cm and Height > 2 m. One parallel sample was taken along the slope, while the other was taken along the contour. All the cores collected reached the pith of the tree. Tree age was estimated from the oldest core of each tree after measuring the tree rings.

### Measuring of the stem morphological traits

The stem morphological traits (Supplementary Table 3) were quantified with the LiDAR point clouds. Data preprocessing, including splicing, denoising and normalization, was performed in RiSCAN PRO and Cloudcompare. Tree segmentation was performed using a shortest-path algorithm and an accuracy assessment, as described in Tao et al. (2015)^23^. Height was calculated as the difference between the highest and the lowest points in a segmented tree. DBH was calculated by using the Taubin method to fit circles to cloud point slices at 1.3 m^52^. Height under the crown was considered the height of the point that divided the trunk and canopy. CA was considered the maximum cross-sectional area of the whole tree canopy, which was calculated by dividing the canopy into several layers, with the vertices and polygon areas calculated by Graham scanning methods^53^. LAI was calculated with a point cloud slicing-based algorithm demonstrated by Li et al.^54^. First, the point cloud was segmented according to different incident and zenith angles. Then, the gap fraction and clumping index were derived based on the cloud. Finally, LAI was calculated with the Beer-Lambert law based on the gap fraction and clumping index.

Several parameters of the stem morphological traits were extracted through human-computer interactions, as they are too difficult to calculate by computer programs alone when leaves are present. The lengths and counts were calculated for the first-order and second-order branches for each sample tree to obtain values of the Sl/Fl and Sc/Fc, respectively. The number of branches and their diameters at 1 m aboveground were measured and then integrated as the trunk dominant ratio, TDR:

1 $${\mathrm{TDR}} = {\mathrm{num}} \times ({\Sigma}d_{\mathrm{i}}/d_{{\mathrm{max}}})$$

where *d*_i_ is the diameter of the stems, *d*_max_ is the largest stem diameter, and num is the number of stems (Supplementary Fig. 2).

### Measurements of leaf morphological traits

The leaf morphological attributes were obtained by measuring the leaf samples acquired from the plots (Supplementary Table 3). LA and LV were calculated from scanned photos using MATLAB R2014a (MathWorks, America). The samples were dried at 65 °C for 48 h. Leaf dry weight was measured after drying. SLA and LTD were calculated by Eqs. (2) and (3), respectively:

2 $${\mathrm{SLA}} = {\mathrm{LA}}/{\mathrm{leaf}}\,{\mathrm{dry}}\,{\mathrm{weight}}$$

3 $${\mathrm{LTD}} = {\mathrm{leaf}}\,{\mathrm{dry}}\,{\mathrm{weight}}/({\mathrm{LA}} \times {\mathrm{leaf}}\,{\mathrm{thickness}})$$

### Collection of environmental data

Based on recent studies, 13 environmental variables were chosen as potential determinants of tree morphological traits^23,49,55,56^: MAP, CVp, Radiation, MAT, PDSI, PET, AET, BD, Altitude, Slope, Aspect, Age, and Cover.

We extracted the mean monthly temperature and precipitation from 1950 to 2000 with a 1 × 1 km resolution from the WorldCLIM dataset^50^. MAT, MAP, and CV_P_ were further calculated with gridded precipitation data. Radiation was obtained from the Solargis database^57^ as the average horizontal irradiation during 2007 and 2016, with a spatial resolution of 250 × 250 m. Values of the PDSI were collected from the CGD’s climate analysis section^58^. We took the average value from 1981 to 2009 as the PDSI of the plots. AET and PET datasets with spatial resolutions of 0.00833° (~1 km) from the CGIAR-CSI Global-Aridity and Global-PET databases^59^ were used. Soil bulk density data were collected from the Harmonized World Soil Database (version 1.1) shared via the Geographic Data Sharing Infrastructure, Peking University^60^.

### Statistics and reproducibility

First, the individual-level potential allometric relationships between Height, CA, and DBH were established using nonlinear 5% and 95% quantile regressions. The 5^th^ percentile was regarded as the minimum necessary value, while the 95^th^ percentile was regarded as the maximum potential value. The formula used in the nonlinear regression followed the power function used in a series of DGVMs^9–14^. The relationships were compared with the theoretical model used in the DGVMs to determine whether our results showed the pattern commonly adopted in the models. Second, the statistical relationships among the morphological traits were uncovered with two-tailed Pearson correlation and partial correlation tests, and later by PCA and cluster analysis. This allowed us to analyze the interaction between each pair of traits and test whether a general coupling relationship exists among the stem and leaf morphological traits. To better display the results, LTD and TDR were set as negative values in the PCA. For the cluster analysis, Euclidean distance and centroid clustering were adopted. Leaf morphological traits are regarded as the proxy indexes of tree water use strategies (Supplementary Fig. 3). Finally, the environmental determinant of the plant morphological traits was selected using two-tailed Pearson correlation, partial correlation and linear regression tests. All the test variances were assumed to meet the assumption of normality. All the analyses were performed in R software (R Development Core Team, 2009).

Reproducibility was achieved during plot selection, stem morphology measuring and leaf sampling. Three 25 × 25 m plots with different slopes and aspects were established at each study site. Tree allometry traits, Height, CA, and DBH, as well as stem morphological traits, CLR and LAI, were measured for all the individuals in the plots, while TDR, Sl/Fl, and Sc/Fc were measured for at least five well-scanned trees in each plot. In each plot, at least 20 leaves were sampled at each position (i.e., sunny and shady branches at middle and lower height of tree canopy), with a total of 80 leaves sampled per plot. Leaf thickness was measured as the average of 10 leaves.

### Reporting summary

Further information on research design is available in the Nature Research Reporting Summary linked to this article.

## Supplementary information

Supplementary Information

Reporting Summary

## Data Availability

The plot average tree morphological traits and environment data are available in the [Peking University Open Research Data Platform] repository, [10.18170/DVN/7QIQ6W]. Other data are available from the corresponding author on reasonable request.
