# Supplementary material for: Drought-modulated allometric patterns of trees in semi-arid forests
Source: Commun Biol. 2020 Jul 30;3:405. doi: 10.1038/s42003-020-01144-4 (PMC7393108; doi:10.1038/s42003-020-01144-4)
Supplement: Supplementary file 1 — Supplementary Information [file 42003_2020_1144_MOESM1_ESM.pdf]

Supplementary Information for:

Drought-modulated allometric patterns of trees in semi-arid forests

Supplementary Figures

Supplementary Figure 1

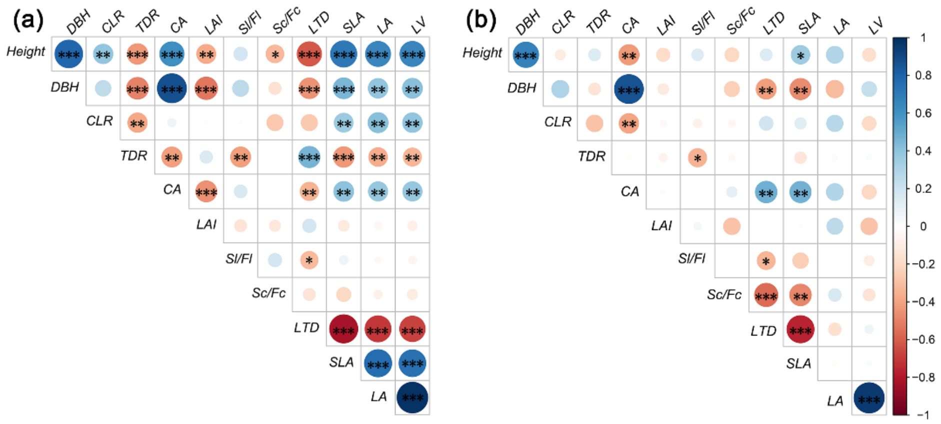

Supplementary Figure 1 (a) Two-tailed Pearson correlation and (b) partial correlation among tree morphological traits.

The size and color of the circles show the correlation coefficients, while the asterisk indicates the significance: *, $P < 0.1$; **, $P < 0.05$; ***, $P < 0.01$. For abbreviations, plant height (*Height*), ratio of height under crown and tree height (*CLR*), breast height diameter (*DBH*), trunk dominance ratio (*TDR*), canopy area (*CA*), leaf area index (*LAI*), ratio of second- and first- order branch length (*Sl/Fl*) and count (*Sc/Fc*), leaf area (*LA*), specific leaf area (*SLA*), leaf tissue density (*LTD*), leaf main vein length (*LV*). For a detailed explanation of the plant traits, refer to Supplementary Table 3.

Supplementary Figure 2

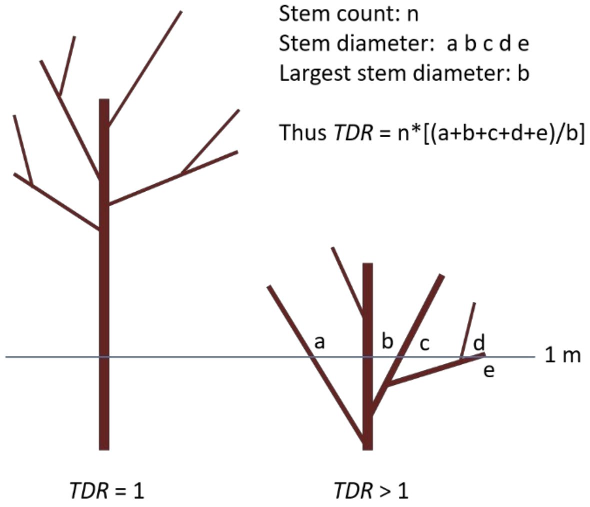

Supplementary Figure 2 A diagrammatic illustration for calculating ‘trunk dominant ratio (*TDR*)’

A greater *TDR* score indicates larger basal ramification.

Supplementary Figure 3

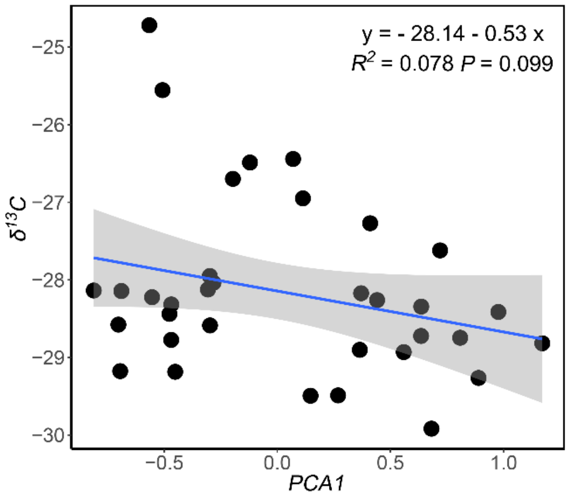

Supplementary Figure 3 The relationship between leaf morphology and leaf water-use efficiency

Principal component analysis (PCA) to reduce the dimensionality of leaf morphological traits showed that the first component (*PCA1*) explained 76.5% of the total variance. Higher *PCA1* score of the plots indicate the leaves to have smaller leaf tissue density (*LTD*) and bigger leaf area (*LA*), greater specific leaf area (*SLA*) and longer leaf vein length (*LV*) averagely. A significant negative linear correlation was found between *PCA1* and leaf $\delta^{13}C$ values (P = 0.099) indicating that leaves with bigger area and lower tissue density tend to have lower $\delta^{13}C$ values.

$\delta^{13}C$ value is an effective proxy index for leaf water use efficiency. A higher value of $\delta^{13}C$ indicates a stronger limitation of stomatal conduction to the intensity of photosynthesis caused by reduced water potential[1, 2, 3]. The formula used for calculating the $\delta^{13}C$ isotope is shown in equation:

$$\delta^{13}C = [\,{}^{13}C/{}^{12}C(sample) - {}^{13}C/{}^{12}C(standard) / {}^{13}C/{}^{12}C(standard)] \times 1000$$

where ${}^{13}C/{}^{12}C$ *(sample)* represents the ${}^{13}C/{}^{12}C$ ratio of our samples and ${}^{13}C/{}^{12}C(standard)$ represents the ratio of the international standard material Pee Dee Belemnite (PDB). The measurement error is 0.45‰ ± 0.08‰[2].

Supplementary Figure 4

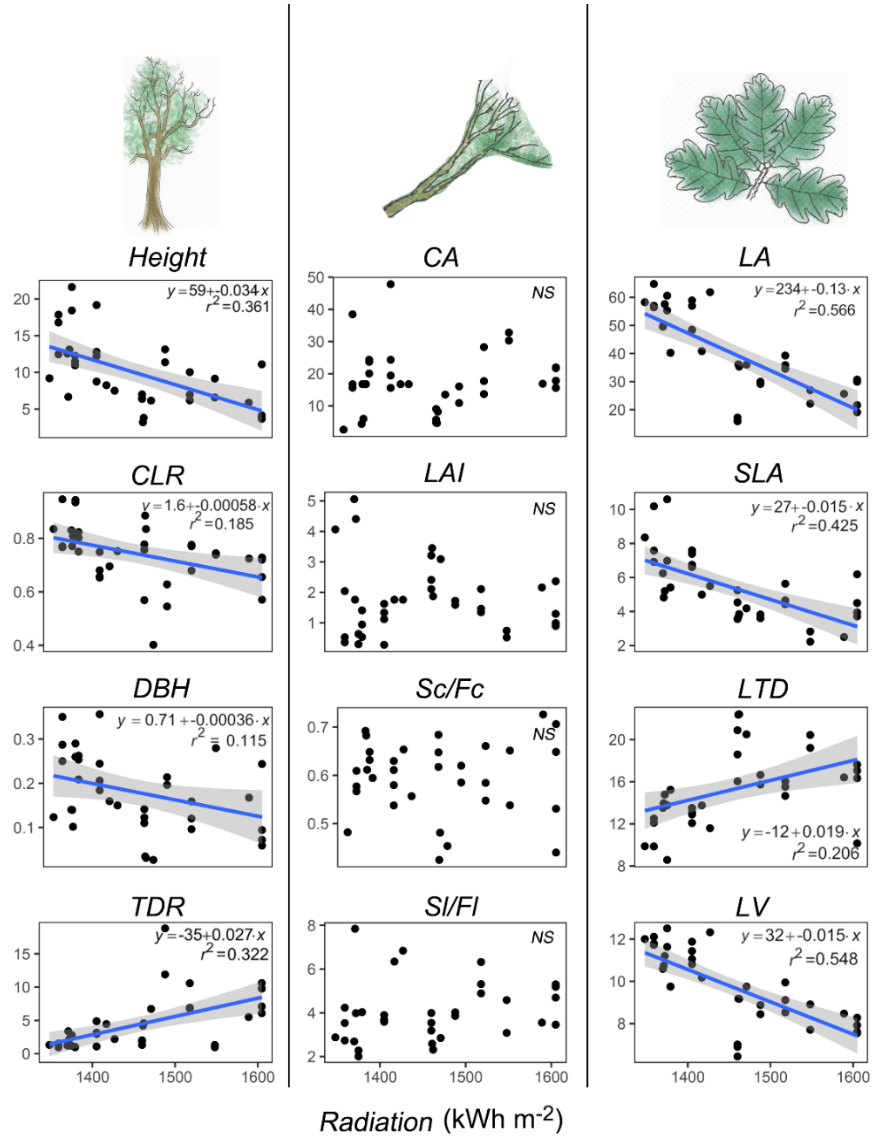

Supplementary Figure 4 Morphological traits variation along the regional solar irradiation (*Radiation*) gradient are shown by scatter plots and linear regression. Each dot represents a site. The blue line represents the linear regression, while the gray shaded region represents the confidence interval of fitting. For abbreviations, plant height (*Height*), ratio of height under crown and tree height (*CLR*), breast height diameter (*DBH*), trunk dominance ratio (*TDR*), canopy area (*CA*), leaf area index (*LAI*), ratio of second- and first- order branch length (*Sl/Fl*) and count (*Sc/Fc*), leaf area (*LA*), specific leaf area (*SLA*), leaf tissue density (*LTD*), leaf main vein length (*LV*). For a detailed explanation of the plant traits, refer to Supplementary Table 3.

• Supplementary Tables

Supplementary Table 1 Partial correlation tests for tree height (*Height*), canopy area (*CA*), diameter at breast height (*DBH*) and the environmental factors that correlated with them.

Controlled variable	Variables	Partial correlation index	Significance
DBH	*MAP-CA*	-0.246	0.154
DBH	*AET-CA*	0.156	0.371
DBH	*Altitude-CA*	-0.324	0.057
Height	*MAP-CA*	-0.391	0.020
Height	*AET-CA*	0.122	0.486
Height	*Altitude-CA*	-0.183	0.292
DBH	*MAP-Height*	0.835	0.000
DBH	*Radiation-Height*	-0.621	0.000
DBH	*AET-Height*	0.497	0.002
DBH	*Altitude-Height*	-0.382	0.023
DBH	*Cover-Height*	0.558	0.001
CA	*MAP-Height*	0.845	0.000
CA	*Radiation-Height*	-0.597	0.000
CA	*AET-Height*	0.487	0.003
CA	*Altitude-Height*	-0.285	0.098
CA	*Cover-Height*	0.643	0.000

Supplementary Table 2 The summary information for each study site, including the location, average tree age, mean annual precipitation (*MAP*) and mean annual temperature (*MAT*).

Sites	Plot ID	Longitude (°N)	Latitude (°E)	Altitude (m a.s.l.)	Slope (°)	Aspect (NE°)	Average tree age (a)	MAT (℃)	MAP (mm)
	BJ1	116.77	42.99	1167	38	215	47		
BJ	BJ2	115.48	40.02	1737	35	165	55	5.1	498
	BJ3	115.48	40.02	1742	37	200	53		
	HY1	105.81	36.14	1951	36	339	65		
HY	HY2	105.81	36.14	2032	25	290	67	5.5	438
	HY3	105.81	36.14	2028	25	110	69		
	HHT1	111.97	41.02	1661	20	265	68		
HHT	HHT2	111.97	41.02	1657	27	220	68	2	355
	HHT3	111.23	40.82	1364	25	270	57		
	JY1	112.07	35.25	1287	10	25	31		
JY	JY2	112.08	35.25	1340	36	340	59	10.5	634
	JY3	112.08	35.25	1341	33	0	44		
	JT1	125.8	43.95	309	24	183	60		
JT	JT2	125.8	43.95	279	29	50	60	4.7	644
	JT3	125.8	43.95	319	23	343	60		
	KQ1	116.77	42.99	1399	5	285	49		
KQ	KQ2	116.77	42.99	1403	5	50	44	1.3	375
	KQ3	116.77	42.99	1400	5	195	43		
	MP1	121.75	37.24	824	25	175	66		
MP	MP2	121.76	37.24	803	30	0	51	11.1	670
	MP3	121.77	37.24	897	25	340	56		
	TJ1	117.55	40.2	829	34	50	54		
TJ	TJ2	117.55	40.2	825	30	98	55	8.5	610
	TJ3	117.55	40.2	825	36	284	69		
	WD1	126.74	43.58	306	40	215	55		
WD	WD2	125.25	41.94	751	35	170	56	4	903
	WD3	125.25	41.94	751	20	330	58		
	WQ1	125.81	43.95	350	33	65	57		
WQ	WQ2	126.74	43.58	319	10	129	58	3.6	710
	WQ3	126.74	43.58	306	5	350	53		
	WC1	117.44	42.17	1298	0	37	80		
WC	WC2	117.44	42.17	1306	32	260	87	1	465
	WC3	117.44	42.17	1358	30	290	72		
	WLH1	121.21	46.65	566	23	345	54		
WLH	WLH2	121.20	46.65	574	27	115	53	1.5	444
	WLH3	121.20	46.65	531	28	107	69		

Supplementary Table 3 Morphological traits chosen in the study.

**Attributes**	***Abbreviation***	**Unit**	**Efficiency**
Plant height	*Height*	m	Allometric growth and biomass accumulation[4, 5]
Ratio of height under crown and tree height	*CLR*	-	Adjusting leaf self-shading extent[6]
Breast height Diameter	*DBH*	cm	Allometric growth[4]
Trunk dominance ratio	*TDR*	-	Redundancy effect of ramification[7]
Ratio of second- and first-order branch length	*Sl/Fl*	-	Maximize tree light capture and biomass production[6]
Ratio of second- and first-order branch count	*Sc/Fc*	-	Maximize tree light capture and biomass production[6]
Leaf area index	*LAI*	m^2 m^{-2}	Plant gas, water, carbon, and energy exchange[7]
Canopy area	*CA*	m^2	Maximize tree light capture and biomass production[6]
Leaf area	*LA*	cm^2	Leaf resource capture[8]
Specific leaf area	*SLA*	cm^2 g^{-1}	Leaf resource capture[8]
Leaf tissue density	*LTD*	g cm^{-3}	Leaf resource capture[8]
Leaf main vein length	*LV*	cm	Leaf resource capture[8]

Supplementary References

1. Farquhar, G. D., Oleary, M. H. & Berry, J. A. On the relationship between carbon isotope discrimination and the inter-cellular carbon-dioxide concentration in leaves. *Aust. J. Plant Physiol.* **9,** 121-137 (1982).
2. Qiu, S., Liu, H., Zhao, F. & Liu, X. Inconsistent changes of biomass and species richness along a precipitation gradient in temperate steppe. *J. Arid Environ.* **132,** 42-48 (2016).
3. Poorter, H. & Farquhar, G. D. Transpiration, intercellular carbon-dioxide concentration and carbon-isotope discrimination of 24 wild-species differing in relative growth-rate. *Aust. J. Plant Physiol.* **21,** 507-516 (1994).
4. Sumida, A., Miyaura, T. & Torii, H. Relationships of tree height and diameter at breast height revisited: analyses of stem growth using 20-year data of an even-aged Chamaecyparis obtusa stand. *Tree Physiol.* **33**, 106-118 (2013).
5. Tao, S. L., Guo, Q. H., Li, C., Wang, Z. H. & Fang, J. Y. Global patterns and determinants of forest canopy height. *Ecology* **97**, 3265-3270 (2016).
6. Sterck, F. J., Bongers, F. & Newbery, D. M. Tree architecture in a Bornean lowland rain forest: intraspecific and interspecific patterns. *Plant Ecol.* **153**, 279-292 (2001).
7. Hernandez-Calderon, E., Mendez-Alonzo, R., Martinez-Cruz, J., Gonzalez-Rodriguez, A. & Oyama, K. Altitudinal changes in tree leaf and stem functional diversity in a semi-tropical mountain. *J. Veg. Sci.* **25**, 955-966 (2014).
8. Baraloto, C. *et al.* Decoupled leaf and stem economics in rain forest trees. *Ecol. Lett.* **13**, 1338-1347 (2010).
