# Supplementary material for: Drought-modulated allometric patterns of trees in semi-arid forests
Source: Commun Biol. 2020 Jul 30;3:405. doi: 10.1038/s42003-020-01144-4 (PMC7393108; doi:10.1038/s42003-020-01144-4)
Supplement: Supplementary file 2 — Reporting Summary [file 42003_2020_1144_MOESM2_ESM.pdf]

nature research

Corresponding author(s): Jingyu Dai, Hongyan Liu

Last updated by author(s): Jul 2, 2020

Reporting Summary

Nature Research wishes to improve the reproducibility of the work that we publish. This form provides structure for consistency and transparency in reporting. For further information on Nature Research policies, see Authors & Referees and the Editorial Policy Checklist.

Statistics

For all statistical analyses, confirm that the following items are present in the figure legend, table legend, main text, or Methods section.

n/a	Confirmed	
☐	☒	The exact sample size (*n*) for each experimental group/condition, given as a discrete number and unit of measurement
☐	☒	A statement on whether measurements were taken from distinct samples or whether the same sample was measured repeatedly
☐	☒	The statistical test(s) used AND whether they are one- or two-sided<br>*Only common tests should be described solely by name; describe more complex techniques in the Methods section.*
☐	☒	A description of all covariates tested
☐	☒	A description of any assumptions or corrections, such as tests of normality and adjustment for multiple comparisons
☐	☒	A full description of the statistical parameters including central tendency (e.g. means) or other basic estimates (e.g. regression coefficient) AND variation (e.g. standard deviation) or associated estimates of uncertainty (e.g. confidence intervals)
☐	☒	For null hypothesis testing, the test statistic (e.g. *F*, *t*, *r*) with confidence intervals, effect sizes, degrees of freedom and *P* value noted<br>*Give P values as exact values whenever suitable.*
☒	☐	For Bayesian analysis, information on the choice of priors and Markov chain Monte Carlo settings
☒	☐	For hierarchical and complex designs, identification of the appropriate level for tests and full reporting of outcomes
☐	☒	Estimates of effect sizes (e.g. Cohen's *d*, Pearson's *r*), indicating how they were calculated

Our web collection on statistics for biologists contains articles on many of the points above.

Software and code

Policy information about availability of computer code

Data collection	Point clouds gained by LiDAR were preprocessed in RiSCAN PRO and Cloudcompare. Part of leaf morphological traits were calculated from scanned photos by counting pixel value using MATLAB R2014a. Other trait data's collection needed no special software or codes.
Data analysis	"car","Hmisc","corrgram","vegan","gclus","cluster","mvpart","MVPARTwrap","psych","SparseM","ggplot2","quantreg" packages in R software (R Development Core Team, 2009) and R studio were used during analysis.

For manuscripts utilizing custom algorithms or software that are central to the research but not yet described in published literature, software must be made available to editors/reviewers. We strongly encourage code deposition in a community repository (e.g. GitHub). See the Nature Research guidelines for submitting code & software for further information.

Data

Policy information about availability of data

All manuscripts must include a data availability statement. This statement should provide the following information, where applicable:

- Accession codes, unique identifiers, or web links for publicly available datasets
- A list of figures that have associated raw data
- A description of any restrictions on data availability

The plot average tree morphological traits and environment data are available in the [Peking University Open Research Data Platform] repository, [https://doi.org/10.18170/DVN/7QIQ6W]. Other data are available from the corresponding author on reasonable request.

Field-specific reporting

Please select the one below that is the best fit for your research. If you are not sure, read the appropriate sections before making your selection.

☐ Life sciences ☐ Behavioural & social sciences ☒ Ecological, evolutionary & environmental sciences

For a reference copy of the document with all sections, see nature.com/documents/nr-reporting-summary-flat.pdf

Ecological, evolutionary & environmental sciences study design

All studies must disclose on these points even when the disclosure is negative.

Study description	Twelve sites were sampled in northern China in 2015 and 2016. Three 25 m × 25 m plots with different slopes and aspects were established at each study site. For stem morphological traits, ten to thirteen stations were established in each plot to ensure that all of the trees were measured. In each plot, we sampled at least 20 leaves at 4 positions respectively for measuring the leaf traits.
Research sample	Quercus mongolica is a typical drought tolerated tree species. It is a widely distributed species associated with warm, dry exposed upslope sites in northeastern Asia, and usually plays an important role in forests there. Q. mongolica has an obvious response to water deficit or other environment variables. Its allometry and morphology varies greatly in different environments. According to existing research, its growth form and distribution has ecological relevant correlations with water acquirement. Thus Q. mongolica is a proper research object to quantify drought-modulated allometry and morphology variations in semi-arid area.
Sampling strategy	Twelve sites along a precipitation gradient with different temperature levels were chosen to sample in northern China in 2015 and 2016. In each plot, we sampled at least 20 leaves at 4 different positions from the randomly selected trees for measuring the leaf traits. For tree allometry and stem morphological traits, ten to thirteen scanning stations were established in each plot to ensure that all of the trees were captured by LiDAR. The sample size is sufficient to show both the climate-forced and random-caused tree allometry and other stem and leaf morphology variations.
Data collection	Fieldworks were finished mainly by Jingyu Dai and Yongcai Wang. Yongcai extracted the allometry data and other stem morphological data from LiDAR point cloud, while Jingyu measured leaf traits in lab. LiDAR data preprocessing, including splicing, denoising and normalization, was performed in RiSCAN PRO and Cloudcompare. After that, tree allometry and stem morphological traits were extracted following the methods recorded in the previous articles. Leaf area and thickness were measured in time to reduce the bias which caused by water loss. Leaf dry mass was measured after the samples been dried at 65°C for 48 hours. Two cores were taken from eight trees per plot to get the average tree age.
Timing and spatial scale	Single-time fieldworks were done during July and August in 2015 and 2016. Study area is located in 12 provinces in the northern China.
Data exclusions	No data was excluded during analysis.
Reproducibility	Reproducibility was achieved during plot selection, stem morphology measuring and leaf sampling. Three 25 m × 25 m plots with different slopes and aspects were established at each study site. Tree allometry traits, Height, CA and DBH, as well as part of other stem morphological traits, including CLR and LAI, were measured for all the individuals in the plots, while TDR, Sl/Fl and Sc/Fc were measured for at least five well-scanned trees in each plot. In each plot, at least 20 leaves were sampled at each position, with a total of 80 leaves sampled per plot. Leaf thickness was measured as the average of 10 leaves.
Randomization	This research is based on observations, thus only involves random sampling, but did not involve random assignment (which applies only to controlled experiments). All sampling procedure is according to standard established approach.
Blinding	No blinding was used during data acquisition and analysis. Because after the research sites confirmed, all the tree individuals and gained data were used in sampling, measuring and analyzing processes, which is randomly enough to eliminate the effects of subjective factors.

Did the study involve field work? ☒ Yes ☐ No

Field work, collection and transport

Field conditions	Mean annual precipitation here is approximately 350–800 mm, and the mean annual temperature is 1.5–14.5 °C according to the WorldCLIM dataset. The study area is located in the ecotone of the temperate monsoon and continental climate. The characteristics of the surface wind among the sites are homogeneous. No obvious evidence of fire, insect attack, cutting or grazing was detected in the plots selected for study.
Location	The study area is situated in the semi-humid to and semi-arid areas in northern China (105-127 °N, 35-47 °E), with elevation range in 300-2000 m.
Access and import/export	Within 36 study plots, 30 of them were located in the unmanaged forests. Other 6 plots were located in the national reserves. Full permissions from the reserve authorities had been acquired before we carried out the fieldworks .
Disturbance	The least amongst of tree ring cores and leaf samples were taken with the preliminaries of meeting the study needs.

Disturbance	Disturbances for both samples and study sites are very limited.

Reporting for specific materials, systems and methods

We require information from authors about some types of materials, experimental systems and methods used in many studies. Here, indicate whether each material, system or method listed is relevant to your study. If you are not sure if a list item applies to your research, read the appropriate section before selecting a response.

Materials & experimental systems

n/a	Involved in the study
☒	☐ Antibodies
☒	☐ Eukaryotic cell lines
☒	☐ Palaeontology
☒	☐ Animals and other organisms
☒	☐ Human research participants
☒	☐ Clinical data

Methods

n/a	Involved in the study
☒	☐ ChIP-seq
☒	☐ Flow cytometry
☒	☐ MRI-based neuroimaging
